# Supplementary material for: Neutral sphingomyelinase 2 regulates inflammatory responses in monocytes/macrophages induced by TNF-α
Source: Sci Rep. 2020 Oct 8;10:16802. doi: 10.1038/s41598-020-73912-5 (PMC7544688; doi:10.1038/s41598-020-73912-5)
Supplement: Supplementary file 1 — Supplementary information. [file 41598_2020_73912_MOESM1_ESM.pdf]

**Supplemental Files**

# Neutral sphingomyelinase 2 regulates inflammatory responses in monocytes/macrophages induced by TNF-α

Fatema Al-Rashed[1], Zunair Ahmad[2], Reeby Thomas[1], Motasem Melhem[3], Ashley J. Snider[4,5], Lina M. Obeid[4], Fahd Al-Mulla[3], Yusuf A. Hannun[4], Rasheed Ahmad[1,*]

[1]Immunology & Microbiology Department, Dasman Diabetes Institute, Dasman 15462, Kuwait

[2]School of Medicine, Royal College of Surgeons in Ireland – Medical University of Bahrain

[3]Genetics and Bioinformatics Department, Dasman Diabetes Institute, Dasman 15462, Kuwait

[4]Stony Brook Cancer Center, Stony Brook University, Stony Brook, NY 11794

[5]Department of Nutritional Sciences, College of Agriculture and Life Sciences, University of Arizona, Tucson, AZ 85721, USA

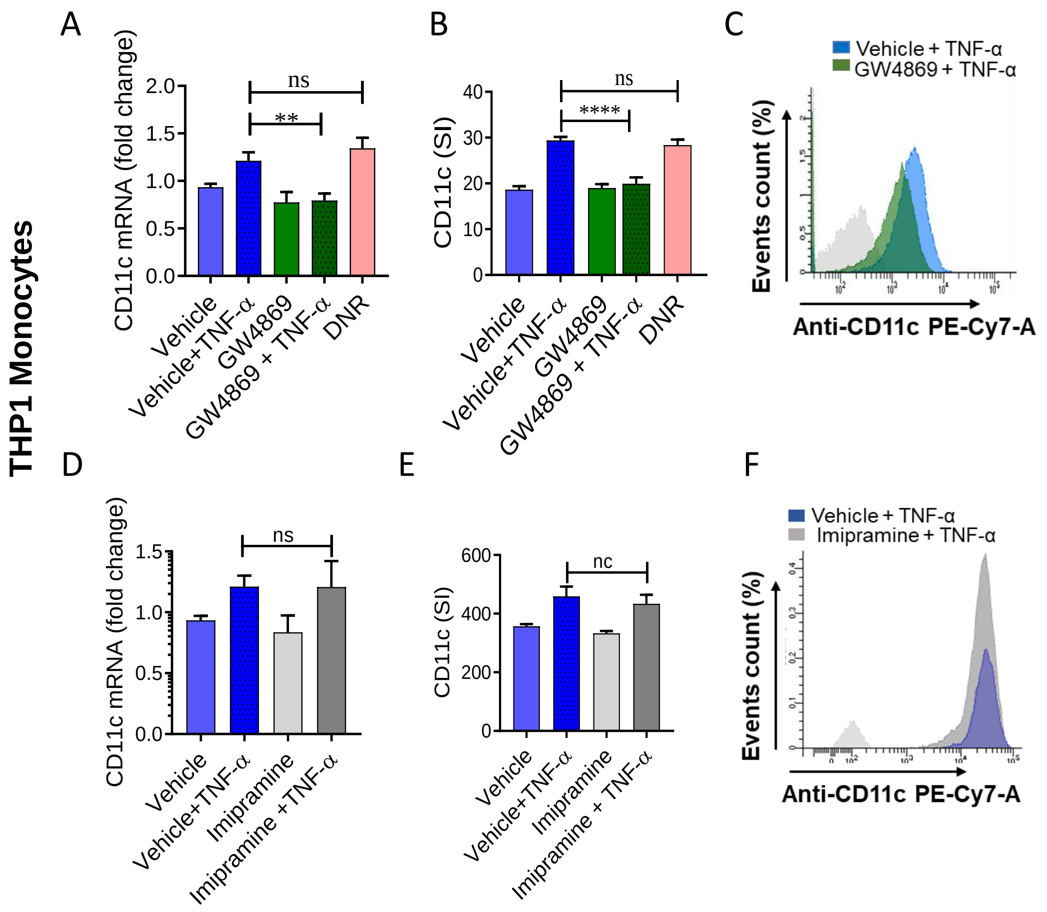


**Figure S1. nSMase inhibition blocks TNF-α mediated CD11c expression in Thp-1**
THP-1 cells were cultured as described in the materials and methods. Cells were pretreated with nSMase inhibitors (GW4869: 10µM), nSMase agonist (DNR: 80nM) or vehicle for 1 hour and then incubated with TNF-α for 2 hours. (**A**) Cells were harvested and mRNA of CD11c was determined by real time RT-PCR. Cells were stained with antibodies against CD14 and CD11b along with matched isotype controls. Surface expression of $CD14^{+}CD11c^{+}$ were assessed by flow cytometry, (**B**) data are presented as a bar graph of mean staining index, and (**C**) representative histogram. THP-1 cells were pre-treated with aSMase inhibitor (lim: 10µM) or vehicle for 1 hour and then incubated with TNF-α for 2 hours. (**D**) mRNA of CD11c and (**E and F**) surface expression of $CD14^{+}CD11c^{+}$ cells were determined. The results obtained from three independent experiments with three replicates of each experiment are shown. All data are expressed as mean ± SEM (n = 3). *p≤0.05, **p≤0.01, ***p≤0.001, ****p≤0.0001 versus vehicle.

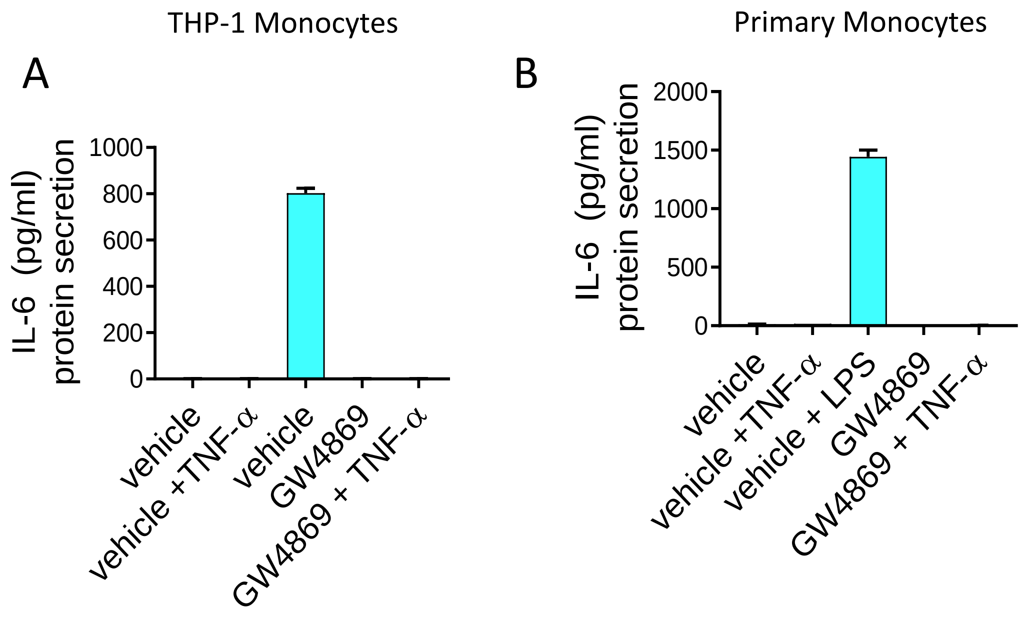


**Figure S2.** TNF-α did not induce IL-6 in human monocytic cells *in vitro*.
Monocytic cells (THP-1 cells; human primary monocytes) were incubated with vehicle, TNF-α (10ng/ml) or LPS (20ng/ml; as a positive control for IL-6 production) for 12 hours. Secreted IL-6 protein in culture media was determined by ELISA. (**A**) IL-6 secreted by THP-1 cells and (B) by primary monocytes. The results obtained from three independent experiments with three replicates of each experiment are shown. All data are expressed as mean ± SEM (n =3).

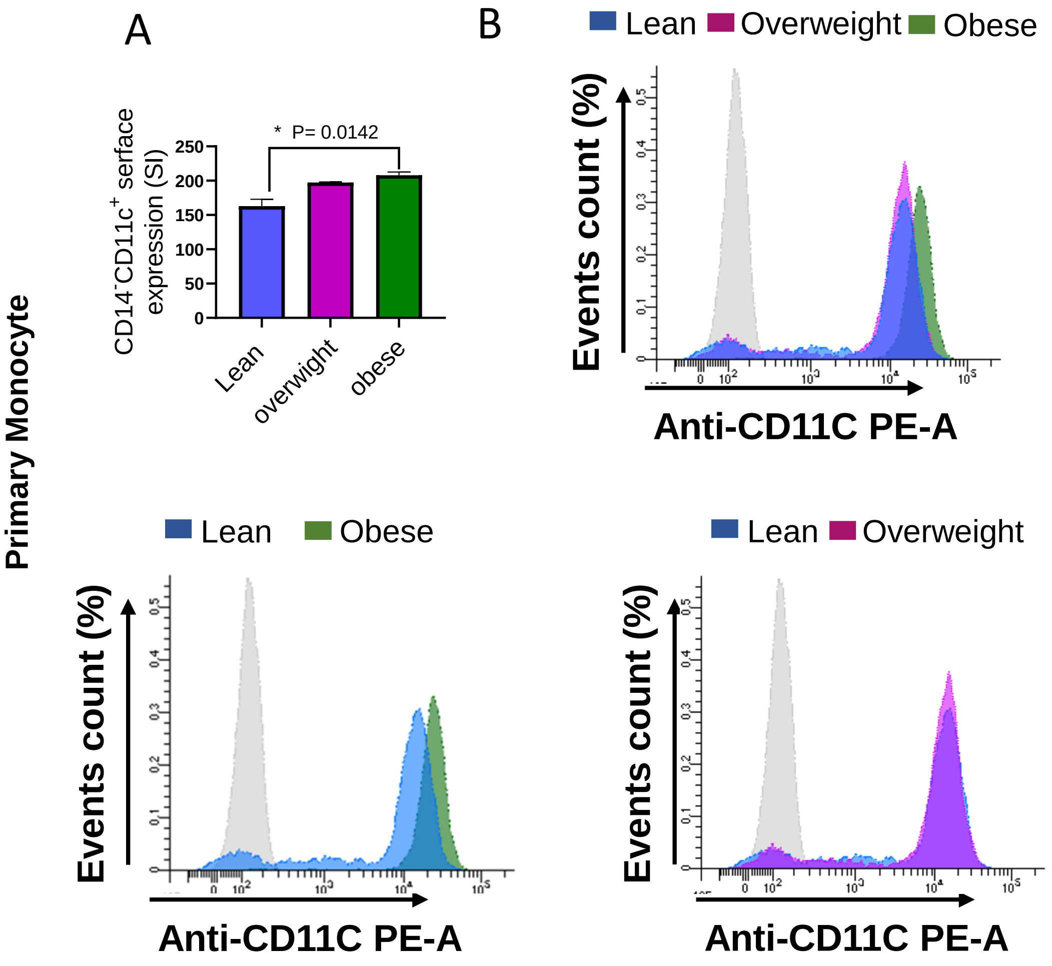


**Figure S3**. CD11c expression on the CD14+ monocytes from Lean, overweight and obese individuals. Cryopreserved PBMCs (Lean 3; overweight 3 ; obese 3) were thawed and washed three times and cultured as described in methods. Cells were stained with antibodies against CD14 and CD11c along with matched isotype controls. Surface expression of CD14+CD11c+ was assessed by flow cytometry, (A) data are presented as a bar graph of mean staining index, and (B) representative histogram.

Gels/Blots-

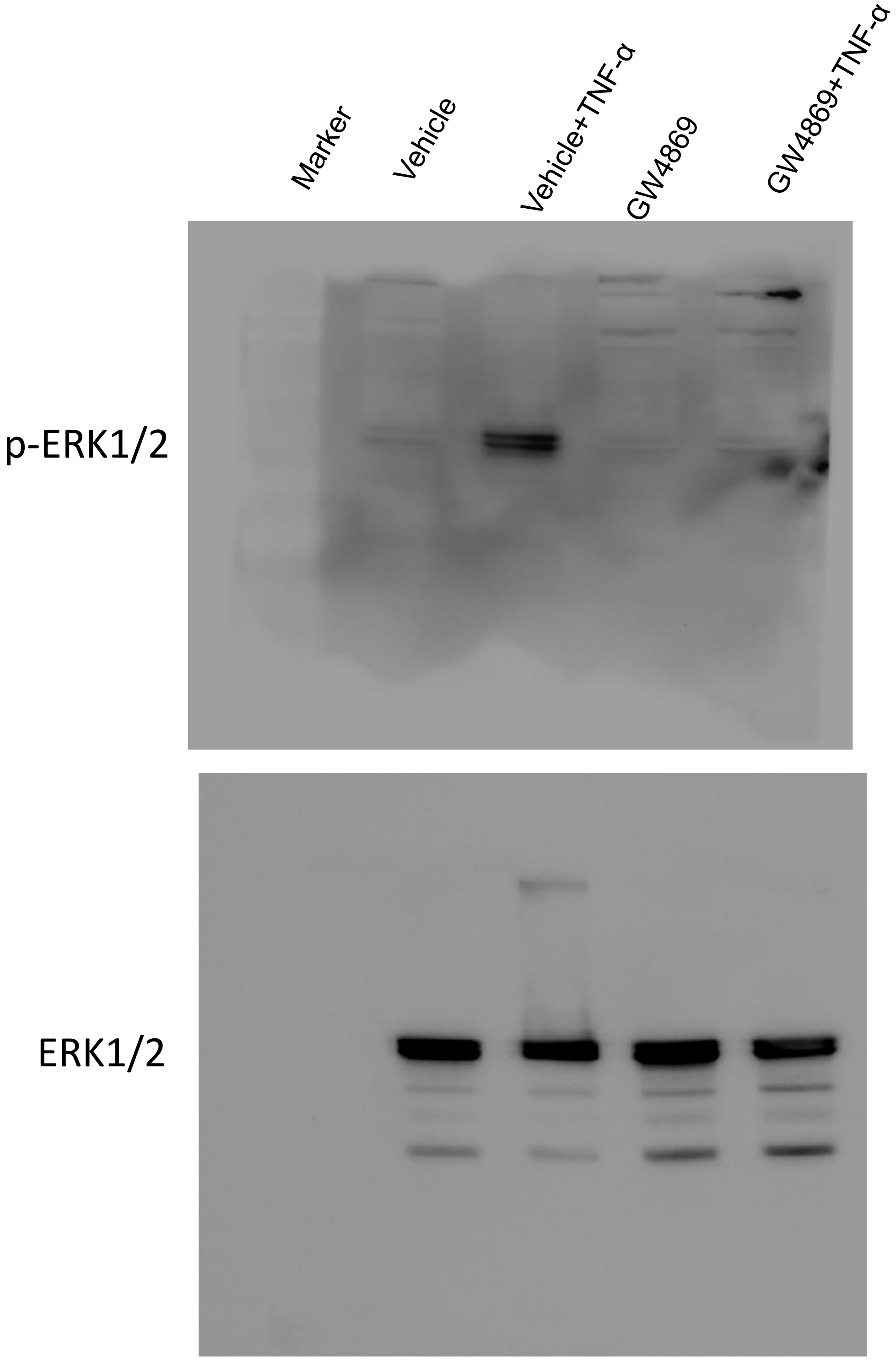
Marker
Vehicle
Vehicle+TNF-α
GW4869
GW4869+TNF-α
p-ERK1/2
ERK1/2

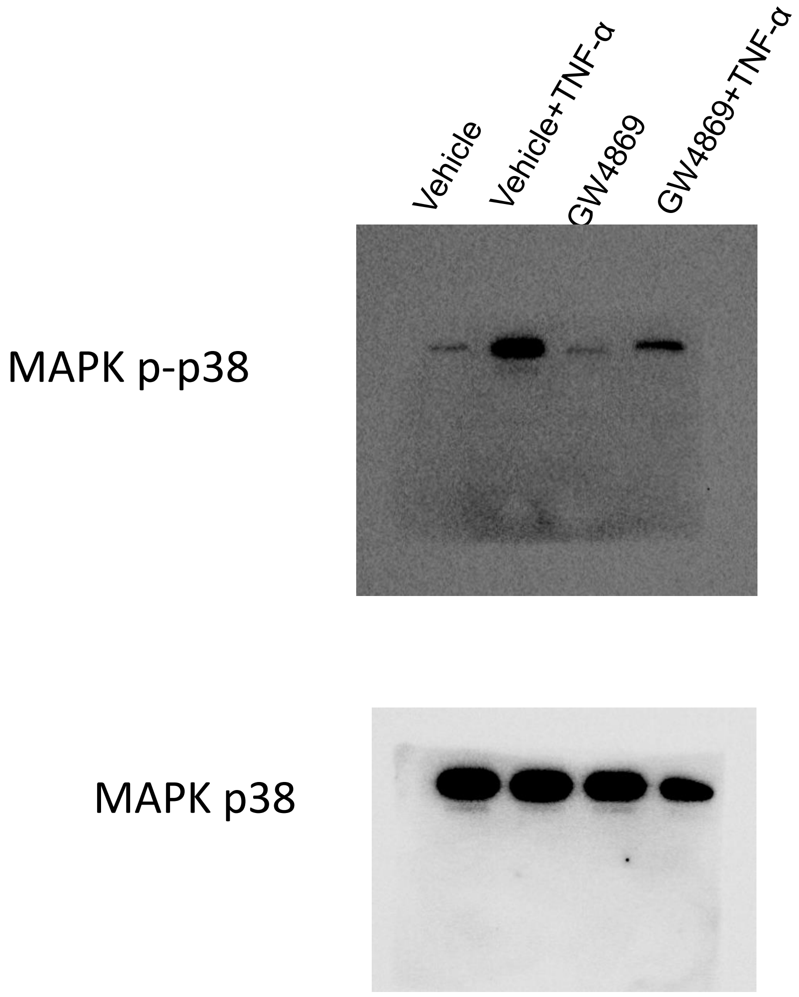
Vehicle
Vehicle+TNF-α
GW4869
GW4869+TNF-α
MAPK p-p38
MAPK p38

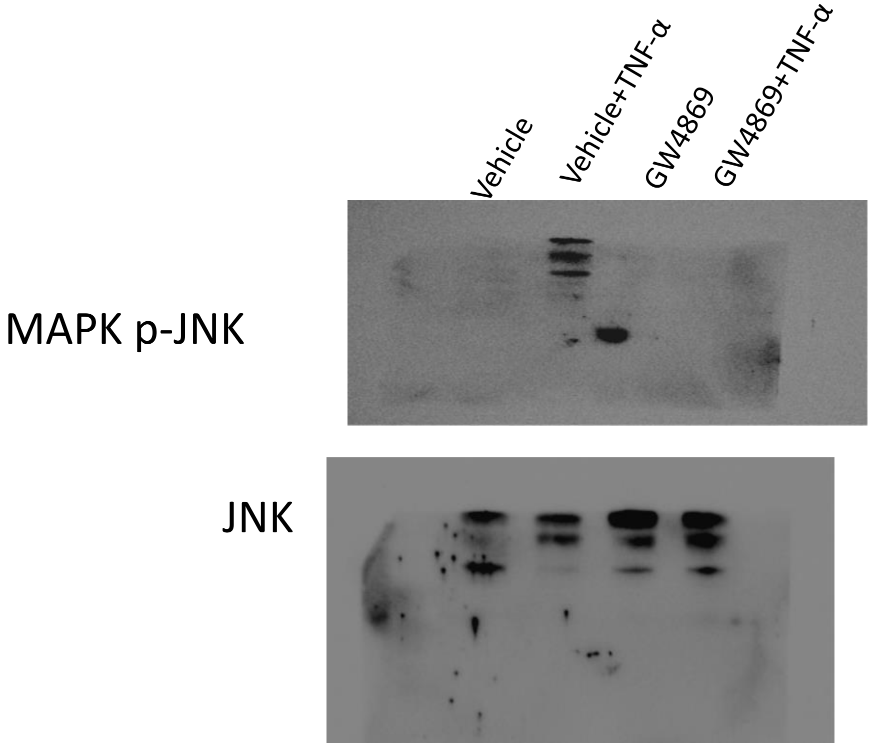
Vehicle
Vehicle+TNF-α
GW4869
GW4869+TNF-α
MAPK p-JNK
JNK

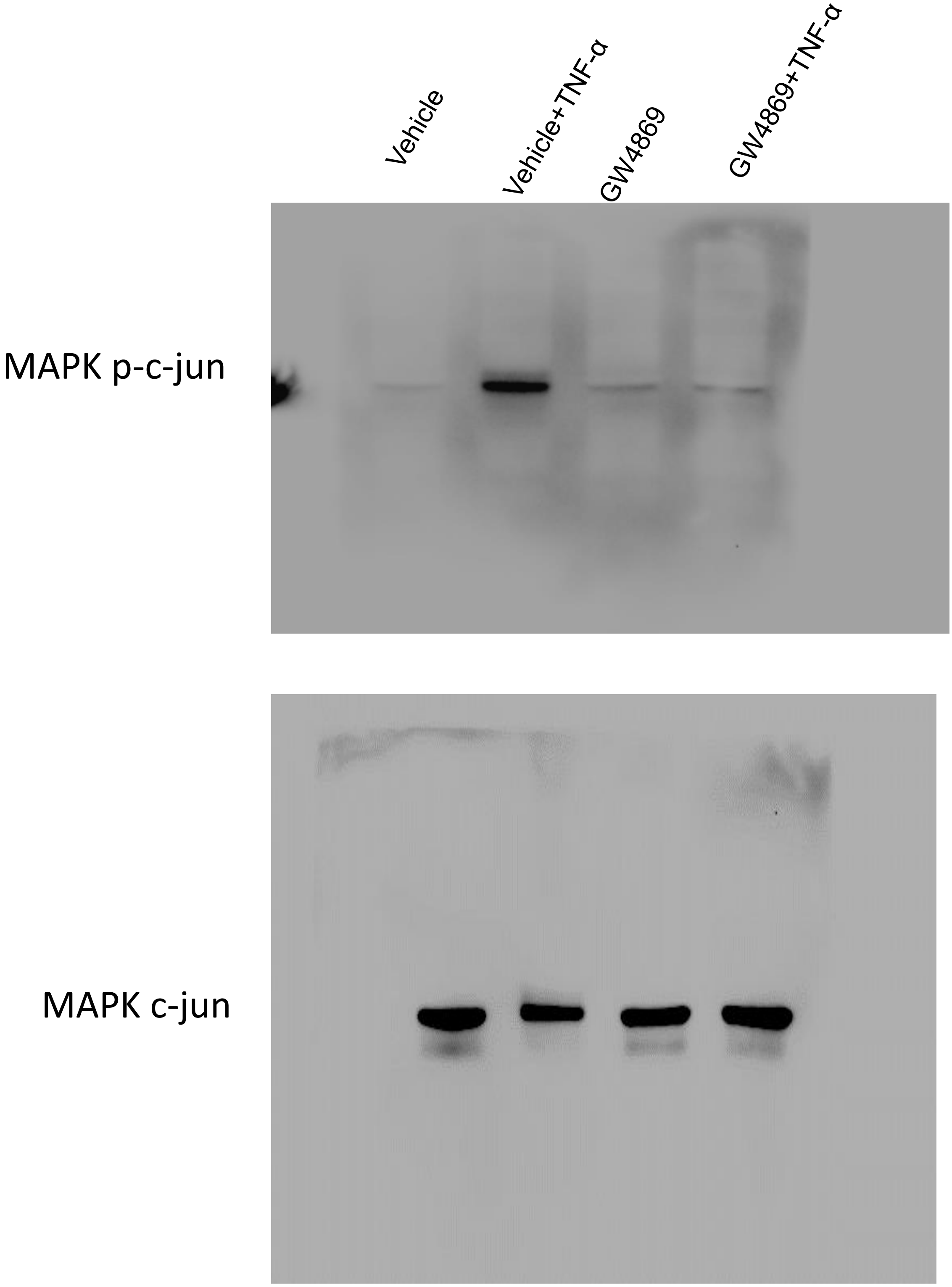
Vehicle
Vehicle+TNF-α
GW4869
GW4869+TNF-α
MAPK p-c-jun
MAPK c-jun

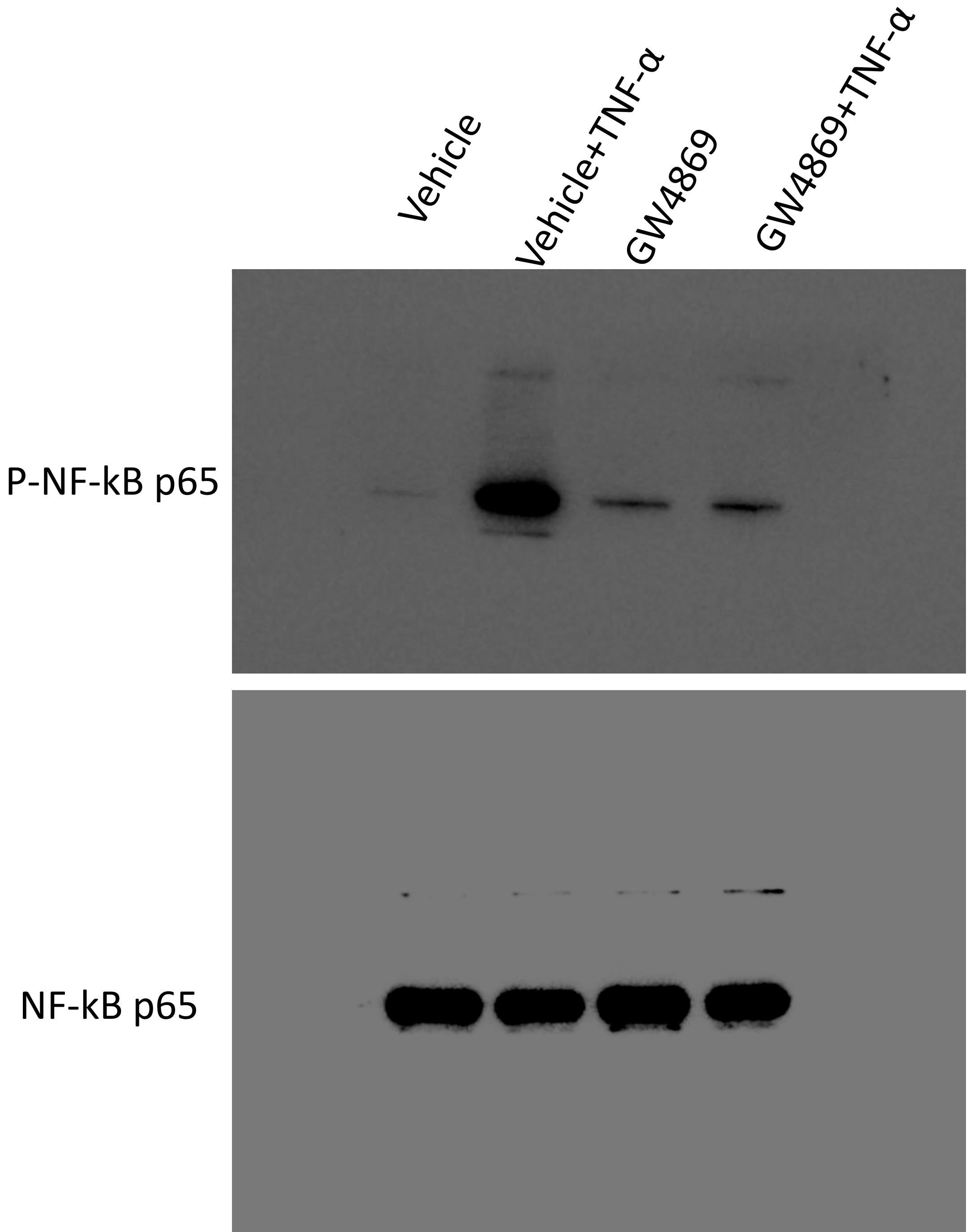

Vehicle
Vehicle+TNF-α
GW4869
GW4869+TNF-α
P-NF-kB p65
NF-kB p65
